# MET-Pyk2 Axis Mediates Acquired Resistance to FGFR Inhibition in Cancer Cells

**DOI:** 10.3389/fonc.2021.633410

**Published:** 2021-04-07

**Authors:** Kamila Kitowska, Monika Gorska-Arcisz, Dima Antoun, Izabela Zarczynska, Dominika Czaplinska, Adrian Szczepaniak, Andrzej C. Skladanowski, Maciej Wieczorek, Aleksandra Stanczak, Monika Skupinska, Rafal Sadej

**Affiliations:** ^1^Department of Molecular Enzymology and Oncology, Intercollegiate Faculty of Biotechnology, University of Gdansk and Medical University of Gdansk, Gdansk, Poland; ^2^Innovative Drugs R&D Department, Celon Pharma, Lomianki/Kielpin, Poland

**Keywords:** FGFR, MET, Pyk2, acquired resistance, cancer

## Abstract

Deregulation of fibroblast growth factor receptors (FGFRs) signaling, as a result of *FGFR* amplification, chromosomal translocation, or mutations, is involved in both initiation and progression of a wide range of human cancers. Clinical data demonstrating the dependence of cancer cells on FGFRs signaling clearly indicate these receptors as the molecular targets of anti-cancer therapies. Despite the increasing number of tyrosine kinase inhibitors (TKIs) being investigated in clinical trials, acquired resistance to these drugs poses a serious therapeutic problem. In this study, we focused on a novel pan-FGFR inhibitor—CPL304110, currently being investigated in phase I clinical trials in adults with advanced solid malignancies. We analyzed the sensitivity of 17 cell lines derived from cancers with aberrant FGFR signaling, i.e. non-small cell lung cancer, gastric and bladder cancer to CPL304110. In order to explore the mechanism of acquired resistance to this FGFR inhibitor, we developed from sensitive cell lines their variants resistant to CPL304110. Herein, for the first time we revealed that the process of acquired resistance to the novel FGFR inhibitor was associated with increased expression of MET in lung, gastric, and bladder cancer cells. Overexpression of MET in NCI-H1703, SNU-16, RT-112 cells as well as treatment with HGF resulted in the impaired response to inhibition of FGFR activity. Moreover, we demonstrated that cells with acquired resistance to FGFR inhibitor as well as cells overexpressing MET displayed enhanced migratory abilities what was accompanied with increased levels of Pyk2 expression. Importantly, inhibition of both MET and Pyk2 activity restored sensitivity to FGFR inhibition in these cells. Our results demonstrate that the HGF/MET-Pyk2 signaling axis confers resistance to the novel FGFR inhibitor, and this mechanism is common for lung, gastric, and bladder cancer cells. Our study suggests that targeting of MET/Pyk2 could be an approach to overcome resistance to FGFR inhibition.

## Introduction

The fibroblast growth factor receptor family comprises four (FGFR1-4) receptor tyrosine kinases (RTKs) involved in the regulation of many downstream effectors including mitogen-activated protein kinase (MAPK), phosphoinositide 3-kinase (PI3K)/Akt, or STATs, that are crucial for cell proliferation, survival, differentiation, and motility (1, 2). Taking into account the significance of these processes in tumorigenesis, aberrant FGFR signaling has been shown to play an essential role in cancer cell survival, proliferation, as well as tumor neoangiogenesis (3). Amplification of *FGFR* genes, chromosomal translocation, and gain-of-function mutations could result either in constitutive ligand-independent FGFR activation or enhanced ligand-dependent signaling, whereby, both processes have been demonstrated to be involved in initiation and progression of a wide range of human cancers (4–6). *FGFR1* amplification has been identified in 10–20% of patients with non-small cell lung cancer whereas 4–10% of primary gastric cancers and approximately 75% of bladder cancers harbor *FGFR2* amplification and FGFR3 mutations, respectively (7–10). These clinical data demonstrate the dependence of cancer cells on FGFR signaling and suggest an urgent requirement of molecular therapies targeting FGFR activity. Several tyrosine kinase inhibitors (TKIs) of FGFRs are currently being investigated in anticancer clinical trials (NCT03344536, NCT02465060, NCT02052778, NCT02393248). However, most small-molecule inhibitors display either limited selectivity for FGFR or significant activity against other RTKs, such as vascular endothelial growth factor (VEGFR) or platelet-derived growth factor (PDGFR) (11–13). Therefore, the discovery of FGFR inhibitors has become a rapidly growing research area (14). According to the mechanism of inhibitor activity, selective FGFR inhibitors either belong to the group of molecules that reversibly occupy the ATP-binding pocket or the group that irreversibly bind covalently to a specific cysteine residue within the receptor kinase domain. As a result of selective FGFR inhibitors’ action phosphorylation of FGFRs and their direct downstream targets, i.e. Fibroblast Growth Factor Receptor Substrate 2-α (FRS2-α) and phospholipase C-γ-1 (PLC-γ-1), is reduced. Despite outstanding progress in the field of targeted therapies and the increasing number of selective inhibitors in clinical trials, adverse effects and development of resistance to applied drugs continue to be serious clinical issues. Only several preclinical and clinical studies investigated the mechanism of acquired resistance to FGFR inhibitors. Secretome screening studies revealed that in multiple cancer cell lines initially dependent on FGFR signaling, activation of the respective alternative kinase could rescue growth upon FGFR inhibitor treatment (15). *In vitro* studies on lung cancer cells showed that decrease in phosphorylation level of extracellular signal-regulated kinase (ERK) 1/2 and Akt mediated by FGFR inhibition with AZD4547 or BAY1163877 was hampered by MET activation (16). Research performed in bladder cancer cell lines revealed a rapid induction of HER2 and HER3 in cells treated with the FGFR inhibitor, BGJ398, thus suggesting that a switch from FGFR- to HER2/3-dependency can compensate FGFR inhibition (17). Protein kinase C (PKC)-mediated phosphorylation of GSK-3β which led to activation of downstream pro-survival proteins was identified as a mechanism of resistance to AZD4547 in gastric cancer cell lines (18). These data indicate the complexity of acquired resistance to FGFR inhibition and pose a significant challenge to identify common pathways responsible for resistance in various cancers.

In this study, we focused on a novel pan-FGFR inhibitor, CPL304110 (Celon Pharma, Poland), which currently has been investigated in phase I clinical trial in adults with advanced solid malignancies (NCT04149691) (19). We analyzed sensitivity to CPL304110 in a panel of cell lines derived from cancers displaying aberrant FGFR signaling, i.e. non-small cell lung cancer, gastric and bladder cancers (20–26). We developed cell line variants resistant to CPL304110 in order to explore a mechanism of acquired resistance. We found that the process was associated with increased expression of MET in lung, bladder, and gastric cancer cells. In addition, ectopic overexpression of MET resulted in impaired cell response to FGFR inhibition. Both, CPL304110-resistant and MET-overexpressing cells were also found to have increased level of Pyk2. Inhibition of MET and Pyk2 restored sensitivity of resistant cells to FGFR inhibition. These results suggest that the MET/Pyk2 axis induces acquired resistance to FGFR inhibition in cancer cells.

## Materials and Methods

### Cell Lines, Antibodies, and Reagents

DMS114, NCI-H1581, NCI-H1703, NCI-H2170, NCI-H520, SNU-1, SNU-5, SNU-16, SW780, and AGS cell lines were obtained from ATCC. 639V, T24, KATO-III, UM-UC-3, UM-UC-14, UM-UC-16, and RT-112 cell lines were obtained from ECACC. SW780 and 639V cells were routinely maintained in DMEM; NCI-H1581 cells in DMEM/F12; UM-UC-3, UM-UC-14, UM-UC-16, and RT-112 cells in EMEM; SNU-5 cells in IMDM; DMS114 cells in Waymouth’s MB752/1. NCI-H1703, NCI-H2170, NCI-H520, SNU-1, SNU-16, AGS, T24, and KATO-III cells were maintained in RPMI 1640 medium. All culture media contained 10% FBS and penicillin/streptomycin (100 U/ml/100 μg/ml). Cells were grown at 37°C in a humidified atmosphere of 5% CO_2_, passaged for a maximum of 3–4 months post resuscitation, and regularly tested for mycoplasma contamination. All culture media and corresponding supplements were purchased from Sigma-Aldrich or Biowest. The following antibodies were obtained from Santa Cruz Biotechnology: anti-EGFR (sc-373746), anti-FAK (sc-271116), anti-FGFR1 (sc-57132), anti-FGFR3 (sc-13121), anti-FGFR4 (sc-136988), anti- FRS2-α (sc-17841), anti-Paxillin (sc-365379). The antibody against β-actin (A5316) was obtained from Sigma-Aldrich. All the remaining antibodies were from Cell Signaling Technology: anti-Akt-Tyr473 (#9271) anti-Akt (#92720), anti-EGFR-Tyr1068 (#3777), anti-Erk1/2-Thr202/Tyr204 (#4377), anti-Erk1/2 (#9102), anti-FAK-Tyr397 (#3283), anti-FGFR-Tyr653/654 (#3471), anti-FGFR2 (#23328), anti-FRS2-α-Tyr196 (#3864), anti-FRS2-α-Tyr436 (#3861), anti-HER2 (#4290), anti-HER2-Tyr1448 (#2247), anti-HER3 (#4754), anti-HER3-Tyr1289 (#4791), anti-MET (#4560), anti-MET-Tyr1235/1236 (#3077), anti-PLC-γ-1-Tyr783 (#2821), anti-PLC-γ-1 (#2822), anti-Pyk2-Tyr402 (#3291), anti-Pyk2 (#3292), anti-Paxillin-Tyr118 (#69363), and anti-Src-Tyr416 (#2101), anti-Src (#2105). Hepatocyte growth factor was purchased from PeproTech. Inhibitors: CPL304110 (WO/2014/141015) was provided by Celon Pharma, Poland; capmatinib was purchased from Selleckchem and PF431396 was purchased from Sigma-Aldrich.

### Cell Proliferation Assay

Cell viability was estimated using the 3-(4,5-dimethylthiazol-2-yl)-2,5-diphenyltetrazolium bromide (MTT) colorimetric assay. Cells were seeded in 96-well plates in triplicates and on the following day treated with DMSO or increasing concentrations of CPL304110 for 48 and 96 h. MTT stock solution was added to each well so that the final concentration of MTT in the medium was 0.5 mg/ml. After 2 h of incubation at 37°C, the medium was discarded and MTT formazan was dissolved in DMSO. The absorbance was measured at 590 nm using a microplate reader.

### Culturing Cells in Three-Dimensional Matrigel^®^

Cell growth in three-dimensional Matrigel^®^ (BD Bioscience Matrigel Matrix Growth Factor Reduced) was carried out as previously described (27, 28). Cells were cultured in regular medium or medium containing indicated inhibitor/growth factor. Media were changed every third day. After 14 days of culture cell growth was evaluated by measuring colonies size (at least 100) using ZEISS PrimoVert microscope and ImageJ software.

### Generation of CPL304110-Resistant Cell Lines

To develop resistance to the FGFR inhibitor CPL304110, NCI-H1703, SNU-16, RT-112 cell lines were gradually exposed to increasing concentrations of CPL304110 with starting dose of the inhibitor at 5 nM. Cells were maintained in medium with the inhibitor which was replaced every 72 h. When the growth kinetics of treated cells were similar to wild-type counterpart cell lines, the concentration of CPL304110 was increased until a final concentration of 5 μM was achieved. After 4–6 months of such culture, resistant cells were established and termed accordingly NCI-H1703R, SNU-16R, and RT-112R.

### Stable Overexpression of MET

NCI-H1703/MET↑, SNU-16/MET↑, and RT-112/MET↑ cell lines were generated with a retroviral system based on pBABE-puro TRP-Met vector (Addgene, #10902) (29). To generate cells with stable expression, the plasmids were transfected into HEK293T packaging cells with TurboFect™ (Invitrogen). After 24 h, medium containing virus particles was collected and used to infect host cells in the presence of 5 μg/ml polybrene. Selection with 5 μg/ml puromycine (Sigma-Aldrich) was conducted to obtain resistant cells with stable overexpression of MET. This was confirmed by western blotting.

### Western Blotting Analysis

For western blot analysis, cells were harvested at 60–70% confluency and lysed with Laemmli buffer (2× concentrated) with 2 mM PMSF, 10 μg/ml aprotinin, 10 μg/ml leupeptin, 5 mM EGTA, 1 mM EDTA, 2 mM Na_4_P_2_O_7_, 5 mM NaF, and 5 mM Na_3_VO_4_. Samples with equal amounts of protein were loaded per well, resolved in SDS–PAGE and then transferred onto nitrocellulose membrane. The membranes were blocked for 1 h in 5% skimmed milk and probed with specific primary antibodies at 4°C. Appropriate secondary Alexa Fluor^®^-conjugated antibodies (680 or 790 nm) (Jackson ImmunoResearch, #111-625-144, #715-655-150) and Odyssey^®^ CLx imaging system (LI-COR^®^ Biosciences) were used to detect protein bands.

### Quantitative PCR

RNA was isolated with PureLink RNA MiniKit (Thermofisher) according to the manufacturer’s protocol. Reverse transcription with random hexamer primers was performed with Transcriptor cDNA First Strand Synthesis Kit (Roche). For analysis of *MET* expression TaqMan probes Hs01565584_m1 and TaqMan Universal PCR Master Mix (Thermofisher) were used. Reactions were done in duplicates. Each plate contained an inter-run calibrator, a set of non-template controls and controls for gDNA contamination. Gene expression was calculated using a modified ΔΔC approach.

### Transwell^®^ Migration Assay

Cell migration was assessed as previously described (30). Briefly, indicated cell lines were seeded onto 6-cm plates. Next day, cells were either pre-treated with CPL304110 (1 μM) or PF431396 (100 nM) for 2 h. Subsequently, cells were detached with enzyme-free cell dissociation buffer (Millipore) and 2.5 × 10^4^ cells were resuspended in serum-free medium ± indicated inhibitor. The polycarbonate membranes (8 μm pores, BD Bioscience) of inserts were coated with high concentration Matrigel^®^ (BD Bioscience) diluted in serum-free medium (1:1). Cells were placed in the inner compartment of the Boyden chamber inserts and allowed to migrate towards complete medium (10% FBS) ± indicated inhibitor. After 24 h of incubation, the non-migratory cells were removed using cotton swab. Membranes were mounted on to glass coverslips, migratory cells were stained with DAPI and counted from 20 random fields under AxioVert 200 fluorescent microscope.

### Statistical Analysis

Data are expressed as mean ± SD from at least three independent experiments. Comparative data were analyzed with the unpaired Student’s t-test using the Statistica^™^ software (v.10; StatSoft^®^, Inc., Tulsa, OK, USA). Two-sided p ≤ 0.05 was considered to indicate a statistically significant difference.

## Results

### Analysis of the Anti-Proliferative Effect of the FGFR Inhibitor CPL304110 in Lung, Gastric, and Bladder Cancer Cell Lines

**Figure 1:**
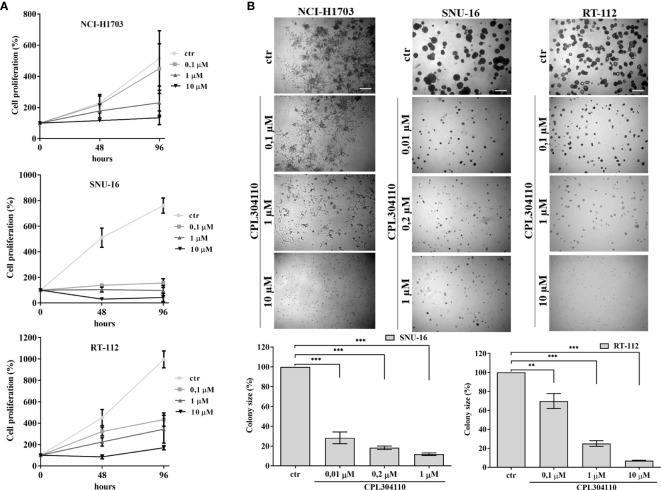
Anti-proliferative effect of CPL304110. **(A)** NCI-H1703, SNU-16, and RT-112 were exposed to the indicated concentrations of the FGFR inhibitor, CPL304110, for 48 and 96 h followed by assessment of cell viability using the MTT assay. Data are expressed as mean ± SD, n = 3. **(B)** Cells were grown in 3D Matrigel^®^ for 14 days in the presence of CPL304110. Representative pictures were taken, colonies were measured and statistically analyzed with ImageJ. Scale bar represents 100 μm. Data are expressed as mean ± SD, **p ≤ 0.01, ***p ≤ 0.001, n = 3.

Aberrant FGFR signaling promotes oncogenic growth in a broad spectrum of solid tumors, indicative that the receptor could be an attractive therapeutic target. Therefore, we examined the anti-proliferative effect of CPL304110, a novel FGFR inhibitor, in a panel of 17 human cell lines from lung, gastric, and bladder cancers. NCI-H1581, NCI-H1703, SNU-16, KATO-III, UM-UC-14, RT-112, and SW780 cells strongly responded to CPL304110 (with IC_50_ ≤1 µM), whereas growth of DMS114, NCI-H2170, NCI-H520, SNU-1, SNU-5, AGS, UM-UC-3, UM-UC-16, 639V, and T24 cells was not significantly affected (**Figures 1A**, **S1A–C**, and **Table S1**). As amplification/overexpression of FGFR1, FGFR2, and FGFR3 contributes to progression of lung, gastric, and bladder cancers, respectively, we verified their level of expression in all analyzed cell lines (4, 24, 31–35). This indicated that CPL304110 exhibits higher potency in the cells expressing elevated level of FGFRs (**Figure S1D**). As FGFR1 is overexpressed in approx. 20% of non-small cell lung cancer (4), the NCI-H1703 non-small cell lung cancer cell line was used for further studies. As a model of gastric and bladder cancers, we respectively used SNU-16 and RT-112 cells, shown to be highly sensitive to FGFR inhibition. The efficacy of CPL304110 in these cells was compared to that of AZD4547, another well-studied FGFR inhibitor. CPL304110 inhibited cell proliferation with a higher potency in comparison to AZD4547, i.e. CPL304110 IC_50_: 1 μM; 0.04 μM and 0.15 μM *vs* AZD4547 IC_50_: 3.4 μM; 0.06 μM and 0.8 μM in NCI-H1703, SNU-16 and RT-112 cells, respectively (Supplementary Data, **Table S2**). The impact of CPL304110 on cell growth was additionally evaluated in 3D cultures in Matrigel^®^ (**Figure 1B**). Since NCI-H1703 cells do not form typical spheroids in 3D Matrigel^®^ and exhibit highly invasive growth, quantitative analysis of the inhibitory effect of CPL304110 was therefore not possible. However, a negative effect of the drug on cell growth was apparent in all tested cell lines. Moreover, we demonstrated a negative effect of CPL304110 on phosphorylation of FGFR and its direct downstream effectors FRS2-α, PLC-γ-1 as well as Akt, Erk1/2 in NCI-H1703, SNU-16, RT-112 cells (**Figure S1E**).

### Development of Resistance to FGFR Inhibitor

**Figure 2:**
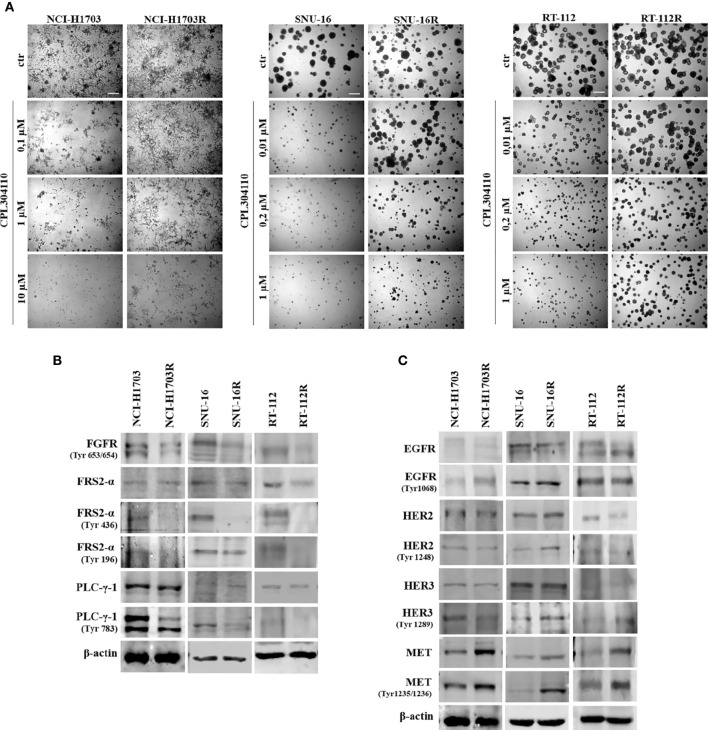
Development of resistance to CPL304110. Resistance to FGFR inhibitor was induced by chronic exposure to CPL304110. **(A)** Response to CPL304110 of parental and resistant cells was evaluated in 3D Matrigel^®^. Representative pictures were taken after 14 days of growth. Scale bar represents 100 µm. Western blot analysis was performed with lysates from parental and resistant cells to assess **(B)** FGFR signaling and **(C)** other RTKs expression/activity level. Experiments were conducted in triplicates.

In order to investigate the mechanisms of acquired resistance to FGFR inhibition, CPL304110-resistant variants of NCI-H1703, SNU-16, and RT-112 cells were developed. Cells were cultured for up to 6 months in the presence of gradually increasing concentrations of CPL304110 in order to develop acquired resistance. Subsequently, sensitivity to CPL304110 was compared with the corresponding parental cell lines. Analysis of 3D growth revealed that NCI-H1703R, SNU-16R, and RT-112R cells demonstrated significantly impaired response to CPL304110 (**Figures 2A**, **S2A**). This was further confirmed by analysis of cell proliferation (**Figure S2B**). In order to determine the mechanism of resistance to FGFR inhibition, we confirmed that the activity of FGFR and its downstream effectors, i.e. Fibroblast Growth Factor Receptor Substrate 2-α (FRS2-α) and phospholipase C-γ-1 (PLC-γ-1), was dramatically impaired in NCI-H1703R, SNU-16R, and RT-112R cells when compared to the corresponding parental cell lines (**Figure 2B**). Since resistance to FGFR inhibition could be initiated by activation of alternative RTKs signaling (15, 36), the expression and activity of EGFR, HER2, HER3, and MET was analyzed in the resistant cell lines (**Figure 2C**). Although there were minor discrepancies in the expression or activity of various RTKs, upregulated MET expression (at both mRNA and protein level) as well as activity was observed in all analyzed resistant cell lines (**Figures 2C**, **S2C**). As a result of this, MET was considered in further studies as a potential inducer of resistance to CPL304110.

### MET Activation Mediates Resistance to FGFR Inhibition

**Figure 3:**
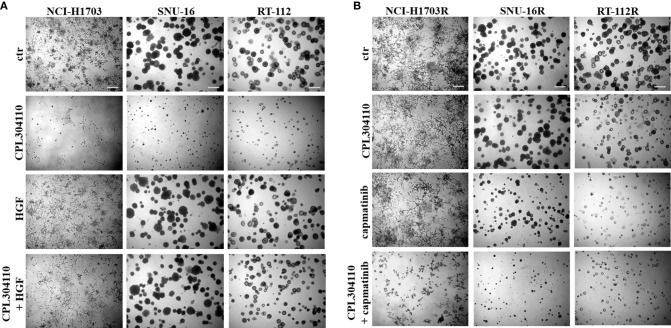
MET activity protects from CPL304110-induced cell growth inhibition. **(A)** NCI-H1703, SNU-16, RT-112 cells were grown in 3D Matrigel^®^ for 14 days in the presence of CPL304110 (1 μM) and/or HGF (50 ng/ml). **(B)** Resistant variants of NCI-H1703, SNU-16, RT-112 cells were grown in 3D Matrigel^®^ for 14 days in the presence of CPL304110 (1 μM) and/or capmatinib - MET inhibitor (5 μM). Representative pictures were taken. Scale bar represents 100 μm, n = 3.

**Figure 4:**
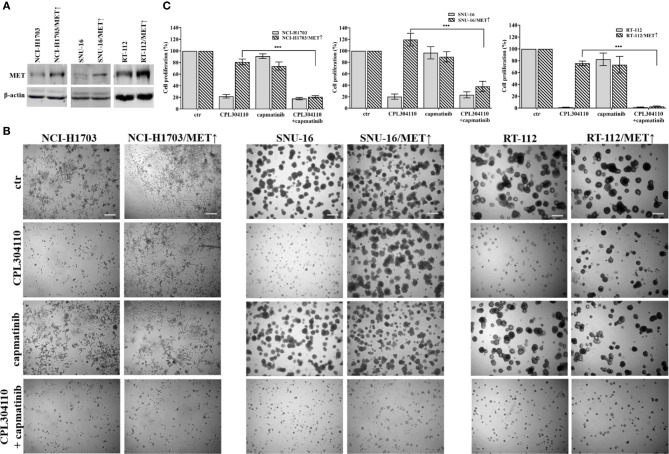
MET overexpression impairs sensitivity to FGFR inhibitor. **(A)** NCI-H1703, SNU-16, RT-112 cells with overexpression of MET were established as described in the materials and methods section. **(B)** NCI-H1703, SNU-16, RT-112 cells, and their variants with MET stable overexpression were grown in 3D Matrigel^®^ for 14 days in the presence of CPL304110 (1 μM) and/or HGF (50 ng/ml). Representative pictures were taken. Scale bar represents 100 μm, n = 3. **(C)** Cell proliferation of either parental or MET-overexpressing cells was assessed using the MTT assay after exposure to CPL304110 (1 μM) and/or capmatinib (5 μM) for 96 h. Data are expressed as mean ± SD, ***p ≤ 0.001, n = 3.

Since we observed that MET phosphorylation was increased in the resistant cell lines, we investigated further whether incubation of the parental cell lines, NCI-H1703, SNU-16, and RT-112 with HGF could counteract CPL304110-dependent inhibition of growth. We observed that although HGF did not significantly promote 3D cell growth, it exerted a strong protective effect against CPL304110 in all tested cell lines (**Figures 3A**, **S3A**). In contrast, inhibition of MET activity with capmatinib (highly sensitive MET inhibitor, FDA approved for NSCLC treatment) restored sensitivity of NCI-H1703R, SNU-16R, and RT-112R cells to CPL304110 (**Figures 3B**, **S3B**). Moreover, stable MET overexpression in NCI-H1703, SNU-16, and RT-112 cells (**Figure 4A**) resulted in strongly impaired sensitivity of these cells to FGFR inhibitor (**Figures 4B, C**). Strikingly, when the combination of CPL304110 and capmatinib was applied in both 3D growth and classical 2D proliferation assays, growth for NCI-H1703/MET↑, SNU-16/MET↑, and RT-112/MET↑ cells was dramatically affected.

### Pyk2 Mediates Migratory Abilities of Cells Resistant to FGFR Inhibitor

**Figure 5:**
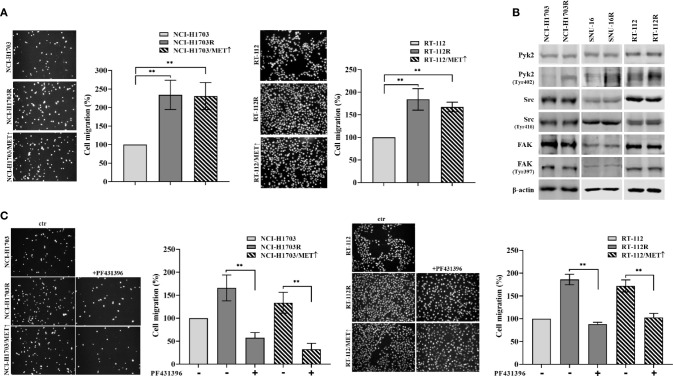
CPL304110-resistant cells exhibit promigratory abilities. **(A)** Parental, CPL304110-resistant, and MET-overexpressing cell variants migrated towards full medium. Data are expressed as mean ± SD, **p ≤ 0.01, n = 3. **(B)** Western blot analysis was performed with lysates from parental and resistant cells to assess phosphorylation levels of Pyk2, Src, and FAK. Experiments were conducted in triplicates. **(C)** The Pyk2 inhibitor PF431396 (100 nM) reduces the migratory abilities of resistant and MET-overexpressing cell variants. Data are expressed as mean ± SD, **p ≤ 0.01, n = 3.

The observed results herein indicated MET involvement in resistance of lung, bladder, and gastric cancer cells to CPL304110. MET activity regulates not only proliferation but also cancer cell motility, invasion, and eventual metastasis (37, 38). Therefore, it was investigated whether MET-dependent resistance to CPL304110 is associated with changes in the migratory abilities of cells. This was verified in transwell migratory assay, which revealed enhanced migration in NCI-H1703R and NCI-H1703/MET↑ cells (2.34-fold and 2.31-fold increase, respectively), as well as in RT-112R and RT-112/MET↑ cells (1.83-fold and 1.67-fold increase, respectively) (**Figure 5A**). Next, we investigated the activity of proteins involved in the regulation of focal adhesions (i.e. Pyk2, FAK, and Src), previously reported to be MET-regulated (39). We explored this in parental and FGFR inhibitor-resistant NCI-H1703, SNU-16, and RT-112 cells. We found that neither FAK nor Src displayed changes in their activity following acquired resistance to CPL304110 (**Figure 5B**), however, upregulated phosphorylation of Pyk2, a non-receptor tyrosine kinase that acts as an integrator of survival, adhesion, and migration, was found in NCI-H1703R, SNU-16R, and RT-112R and in cells with ectopic MET expression (**Figure S4A**) (40). Moreover, MET inhibition led to decrease in Pyk2 phosphorylation in these cells (**Figure S4B**). Next, it was investigated whether inhibition of Pyk2 could deteriorate migratory potential of CPL304110-resistant and MET-overexpressing cells, as we could observe that Pyk2 inhibition resulted in decrease of FAK and paxillin phosphorylation in CPL304110 resistant cells (**Figure S4C**). Application of PF431396-Pyk2 inhibitor resulted in significantly decreased migration of NCI-H1703R (165.94 *vs* 57.49%), RT-112R (179.86 *vs* 88.26%), NCI-H1703/MET↑ (133.55 *vs* 32.62%), and RT-112/MET↑ (171.98 *vs* 102.70%) (**Figure 5C**).

### Pyk2 Mediates MET-Dependent Resistance to FGFR Inhibition

**Figure 6:**
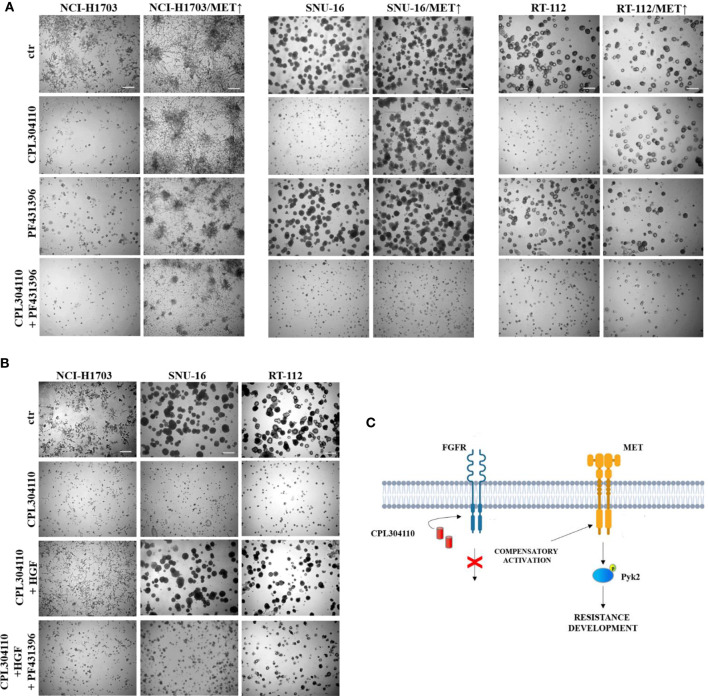
Inhibition of Pyk2 activity restores sensitivity to CPL304110. **(A)** Parental and MET-overexpressing variants of NCI-H1703, SNU-16, RT-112 cells were grown in 3D Matrigel^®^ for 14 days in the presence of CPL304110 (1 μM) and/or Pyk2 inhibitor, PF431396 (100 nM). **(B)** NCI-H1703, SNU-16, RT-112 cells were grown in 3D Matrigel^®^ for 14 days in the presence of CPL304110 (1 μM), HGF (50 ng/ml), and/or Pyk2 inhibitor, PF431396 (100 nM). Representative pictures were taken, scale bar represents 100 μm, n = 3. **(C)** Schematic presentation of the proposed mechanism of acquired resistance to FGFR inhibition.

To further elucidate the functional relevance of Pyk2 in CPL304110-resistance, NCI-H1703/MET↑, SNU-16/MET↑, and RT-112/MET↑ cells were treated with Pyk2 inhibitor (PF431396). Although inhibition of Pyk2 had modest effect on growth of cells overexpressing MET, it re-sensitized them to the FGFR inhibitor (**Figures 6A**, **S5A**). Similar effects were observed in resistant cell variants (**Figure S5B**). It is well known that Pyk2 links numerous signaling pathways and that its activity is induced by heregulin (HRG), epidermal growth factor (EGF), or transforming growth factor-β (TGF-β) (41, 42). Thus, it was elucidated whether HGF/MET→Pyk2 signaling confers resistance to CPL304110. We observed that inhibition of Pyk2 abrogated protective effect of HGF for CPL304110-treated NCI-H1703, SNU-16, and RT-112 cells (**Figures 6B**, **S6**). Collectively, these observations indicate that HGF/MET signaling triggers a Pyk2-mediated mechanism of resistance to FGFR inhibitor that appears to be common in lung, gastric, and bladder cancer cells (**Figure 6C**).

## Discussion

Deregulated FGFR signaling as a result of *FGFR* gene amplification, mutations, or fusions has been identified in various malignancies including lung, gastric, and bladder cancers (43). This provided a strong rationale for development of anti-FGFR therapeutic agents classified as non-selective and selective FGFR inhibitors. Non-selective FGFR TKIs, e.g. nintedanib, lenvatinib, dovitinib, lucitanib, exert a series of toxic effects due to poor target selectivity (13, 44–46). Second generation selective FGFR TKIs have been developed to avoid off-target effects, however, only a few of them entered clinical trials. Studies for AZD4547 and BGJ389 revealed that patients with *FGFR1* amplification (non-small cell lung cancer) or bearing *FGFR3* mutations (bladder cancer) displayed a partial response to the therapy (47, 48). Thus far, erdafitinib and pemigatinib are the first FDA-approved FGFR selective inhibitors for treating urothelial cancer with FGFR2 or FGFR3 alterations, and cholangiocarcinoma with FGFR2 fusions and rearrangements, respectively (49–51). Despite the increasing number of selective FGFR inhibitors, poor clinical response and acquired resistance to these drugs remain the main clinical issue (52, 53).

In the present study, we provide preclinical data for a novel FGFR TKI, CPL304110, that has recently entered phase I of clinical trials in adults with advanced solid malignancies. Our *in vitro* data performed in a panel of 17 human cell lines derived from lung, gastric, bladder cancers showed that CPL304110 inhibits growth of cells displaying elevated FGFR expression with higher potency than AZD4547. Tudrej and colleagues also confirmed higher efficacy of CPL304110 in comparison to AZD4547 in ovarian cancer cells (54). In order to investigate the mechanism of acquired resistance to CPL304110, we generated resistant variants of lung, gastric, and bladder cancer cell lines. We observed that the activity of FGFRs and their direct downstream effectors, i.e. FRS2-α and PLC-γ-1 was abolished in resistant cells and that this was accompanied by upregulation of MET expression. Increased activity of MET has been previously reported to mediate resistance to EGFR inhibitors (e.g. osimertinib) in non-small cell lung cancer *via* EGFR-independent phosphorylation of HER3 and PI3K/Akt activation, providing a bypass pathway in the presence of an EGFR inhibitor (55). In our studies, the stable overexpression of MET in CPL304110 sensitive cells abolished a negative effect of the drug for cell growth, whereas inhibition of MET activity in resistant cells restored sensitivity to CPL304110. These results are in concordance with previously published data, demonstrating that resistance to FGFR inhibitors in *FGFR1*-amplified lung cancer cells can be acquired through MET activation (16, 56). Consistent with our results, HGF-mediated activation of MET has previously been shown to protect RT-112 cells from the effect of FGFR inhibition with BGJ398 (15). Interestingly, Grygielewicz and colleagues showed that the mechanism of resistance to AZD4547, BGJ389, and PD173074 in SNU-16 cells was accompanied with epithelial-mesenchymal transition and impaired activation/expression of RTKs, such as MET, HER2, HER3, or EGFR (35). Activation of HGF/MET signaling was previously proved to promote proliferation, survival, angiogenesis, wound healing, tissue regeneration, scattering, motility, invasion, and branching morphogenesis (57). Herein, we have demonstrated that elevated expression of MET in CPL304110-resistant cells promotes their migration and Pyk2, member of the focal adhesion kinase family, is involved in this process. These results are in concordance with previously published data, showing HGF/MET-mediated migration in small cell lung cancer cell lines with simultaneous Pyk2 phosphorylation on Tyr402 in response to HGF (58). Moreover, Verma and colleagues demonstrated that MET-mediated activation of Pyk2 contributes to metastasis of breast cancer (42, 59). Interestingly, Pyk2 is also considered as an independent prognostic factor for non-small cell lung cancer patients, as high expression and phosphorylation levels of Pyk2 were correlated with poor overall survival (60). Moreover, Pyk2-mediated induction of proliferation in both normal and tumor cells has been widely studied (61, 62) and involves ERK1/2, PI3K-Akt, Wnt/β-catenin activity (63–67).

In conclusion, our study indicates that MET-mediated activation of Pyk2 could confer resistance to CPL304110, a novel FGFR inhibitor and that the mechanism appears to be common in lung, gastric, and bladder cancer cell lines, thus suggesting that targeting of MET→Pyk2 axis could be a therapeutic strategy to overcome resistance to FGFR inhibitors. Further clinical activity evaluation of these pathways would be required to identify patients who are likely to develop resistance to the drug and would therefore benefit from combined therapy.

## Data Availability Statement

The original contributions presented in the study are included in the article/**Supplementary Material**. Further inquiries can be directed to the corresponding authors.

## Author Contributions

KK planned and carried out the experiments and data curation, wrote and edited the manuscript, and prepared all figures. MG-A carried out the experiments and edited manuscript. DA, IZ, DC, and ASz carried out the experiments. MW, ASt, and MS took part in funding acquisition, reviewed and made significant revisions of the manuscript, and guidance of chemical synthesis of CPL304110. ASk reviewed and made revisions of the manuscript. RS took part in funding acquisition, provided direction and guidance throughout the experimental process and preparation of this manuscript. All authors contributed to the article and approved the submitted version.

## Funding

This study was supported within the “CELONKO” project (STRATEGMED2/266776/17/NCBR/2015), co-financed by the Polish National Center of Research and Development, and the pharmaceutical company CelonPharma S.A.

## Conflict of Interest

MW, ASt and MS are employees of Innovative Drugs R&D Department, Celon Pharma (The company responsible for CPL304110 development and synthesis).

The remaining authors declare that the research was conducted in the absence of any commercial or financial relationships that could be construed as a potential conflict of interest.​​​​​​​​​​​​​​​​​​​​
