# Supplementary material for: MET-Pyk2 Axis Mediates Acquired Resistance to FGFR Inhibition in Cancer Cells
Source: Front Oncol. 2021 Apr 7;11:633410. doi: 10.3389/fonc.2021.633410 (PMC8059549; doi:10.3389/fonc.2021.633410)
Supplement: Supplementary file 1 [file DataSheet_1.pdf]

Supplementary Material

Supplementary Figure 1.

A

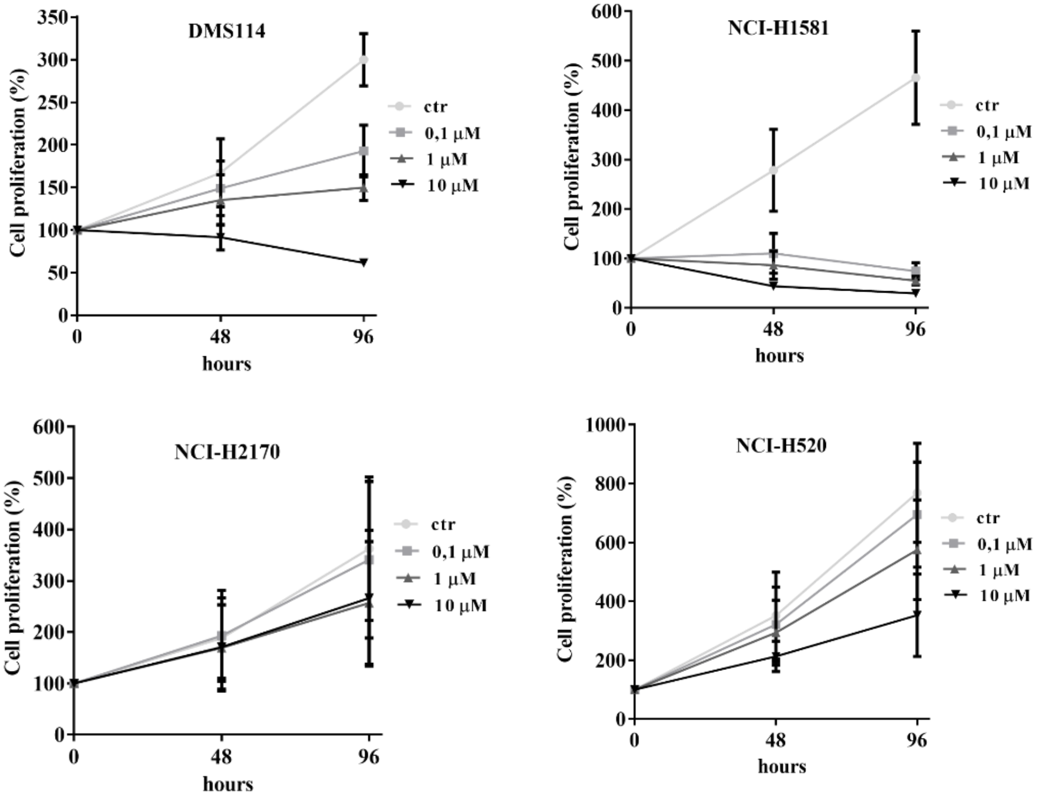

B

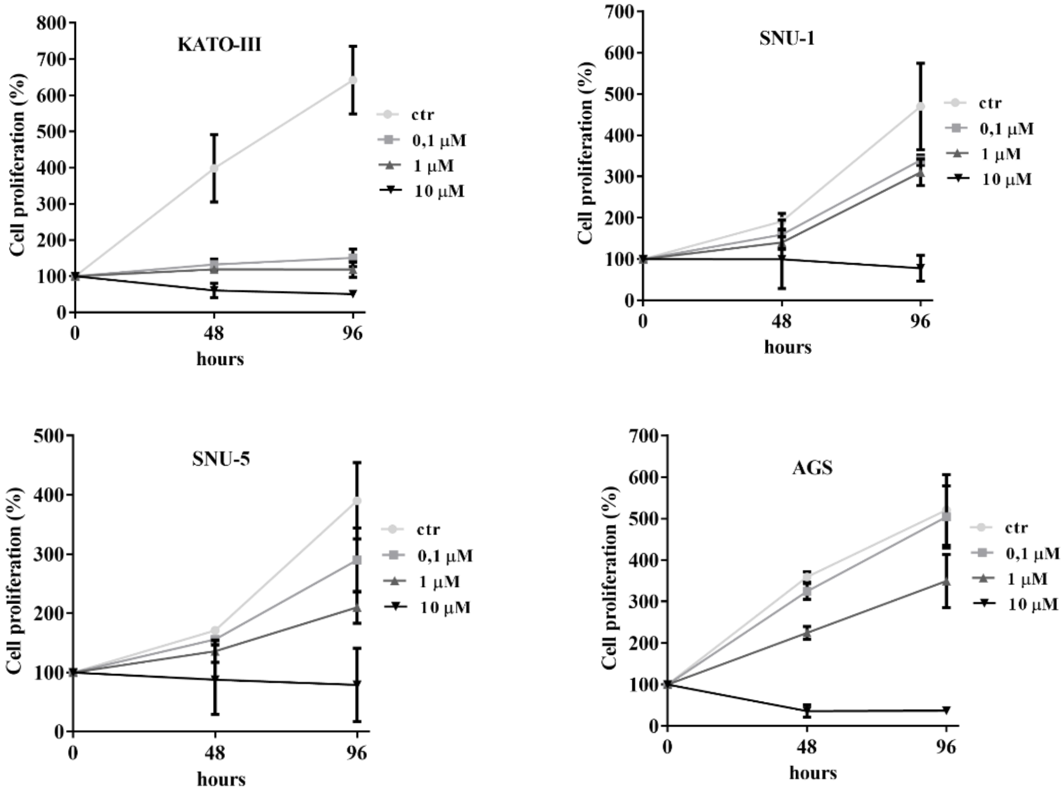

C

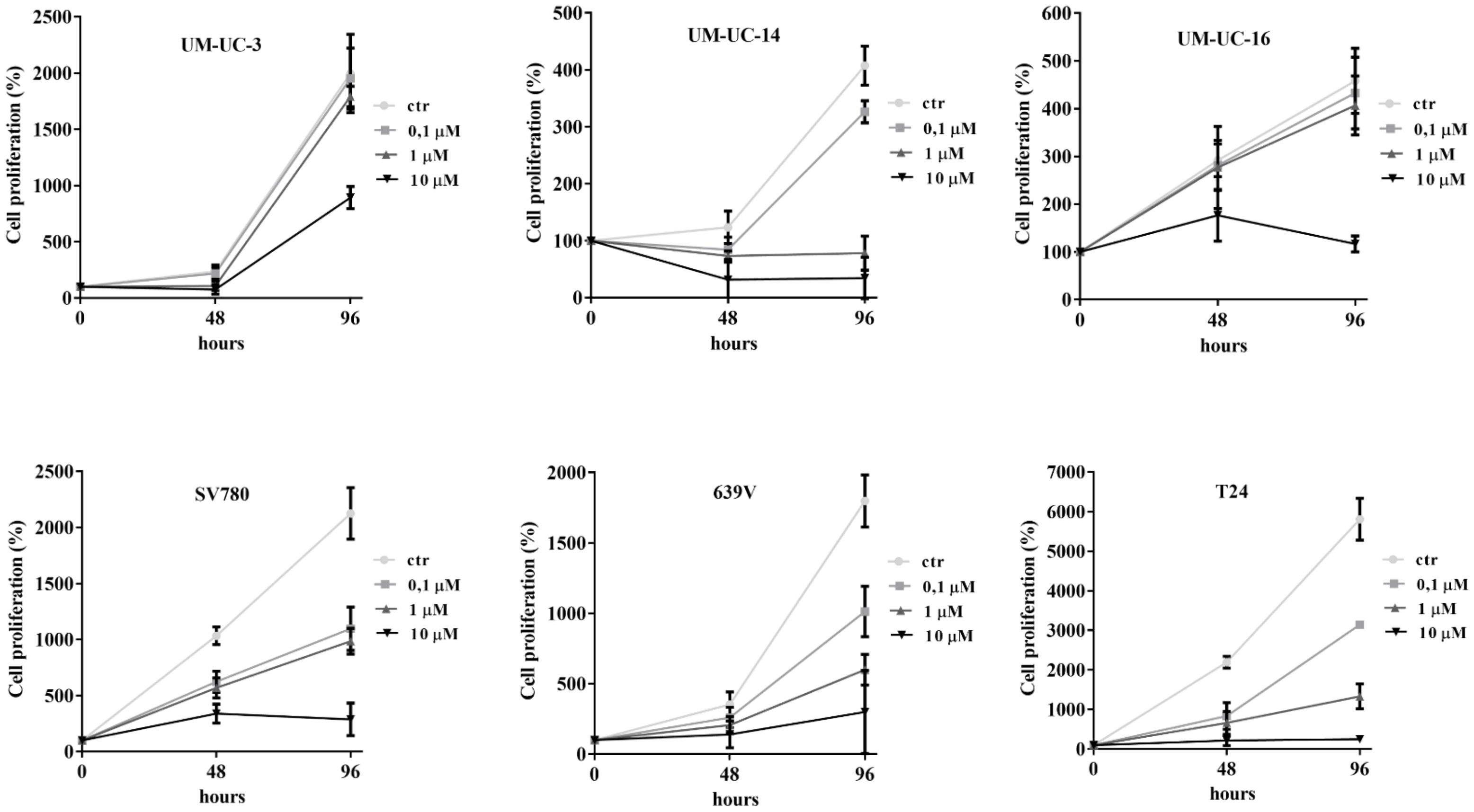
UM-UC-3
UM-UC-14
UM-UC-16
SV780
639V
T24
Cell proliferation (%)
hours
ctr
0,1 µM
1 µM
10 µM

D

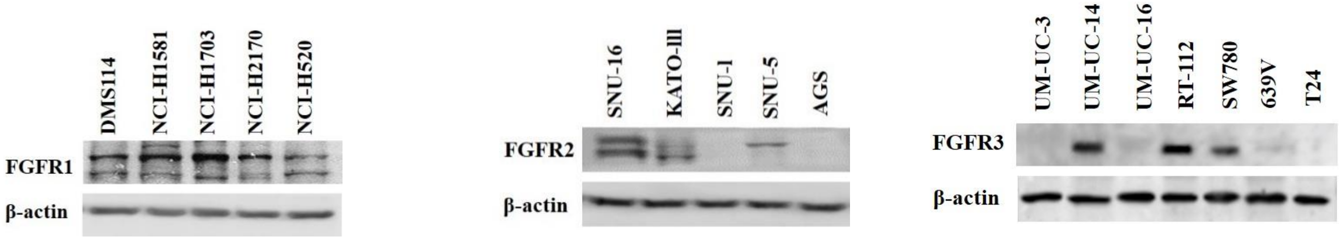
DMS114
NCI-H1581
NCI-H1703
NCI-H2170
NCI-H520
FGFR1
β-actin
SNU-16
KATO-lll
SNU-1
SNU-5
AGS
FGFR2
β-actin
UM-UC-3
UM-UC-14
UM-UC-16
RT-112
SW780
639V
T24
FGFR3
β-actin

E

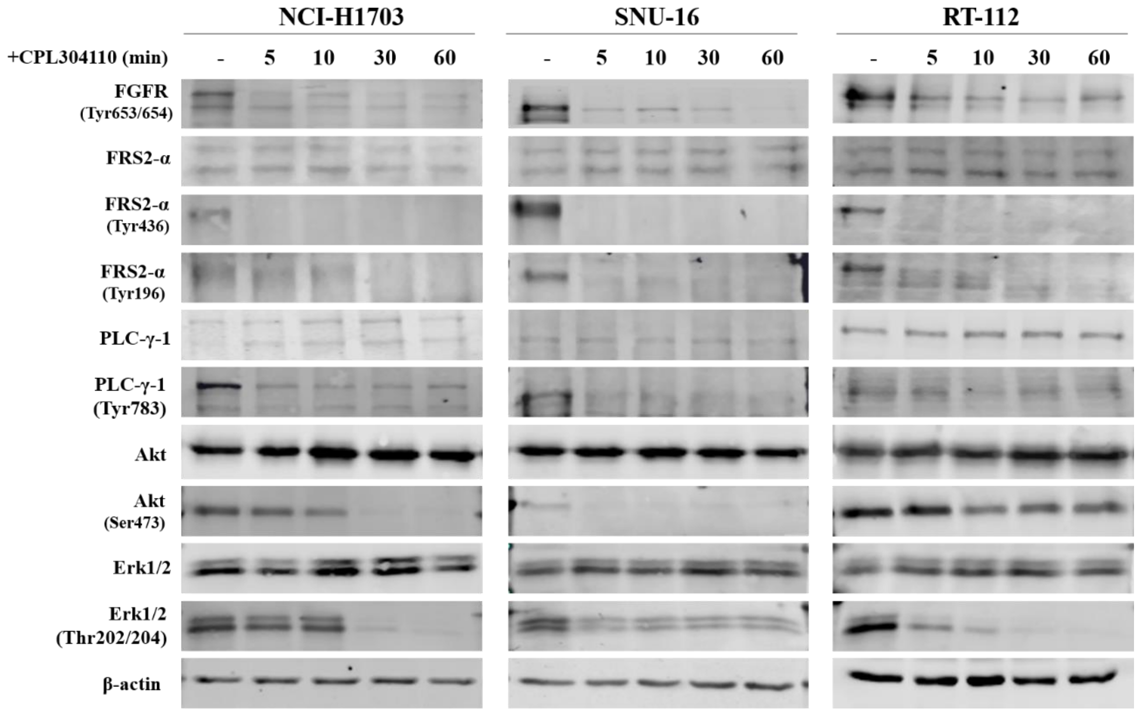

Figure S1. CPL304110 growth-inhibiting activity. Cell viability was assessed using the MTT assay in **(A)** lung cancer cells, **(B)** gastric cancer cells and **(C)** bladder cancer cells, exposed to CPL304110 at the indicated concentrations for 48 h and 96 h. Data are expressed as mean ± SD, n=3. **(D)** Western blot analysis was performed to evaluated expression levels of FGFR1, FGFR2 and FGFR3 in lung, gastric and bladder cancer, respectively. Experiments were conducted in triplicates. **(E)** Western blot analysis was performed to evaluated phosphorylation of FGFR and its downstream effectors in lung, gastric and bladder cancer, respectively. Cells were treated with CPL304110 (1 μM) for indicated time. Experiments were conducted in triplicates.

Supplementary Figure 2.

A

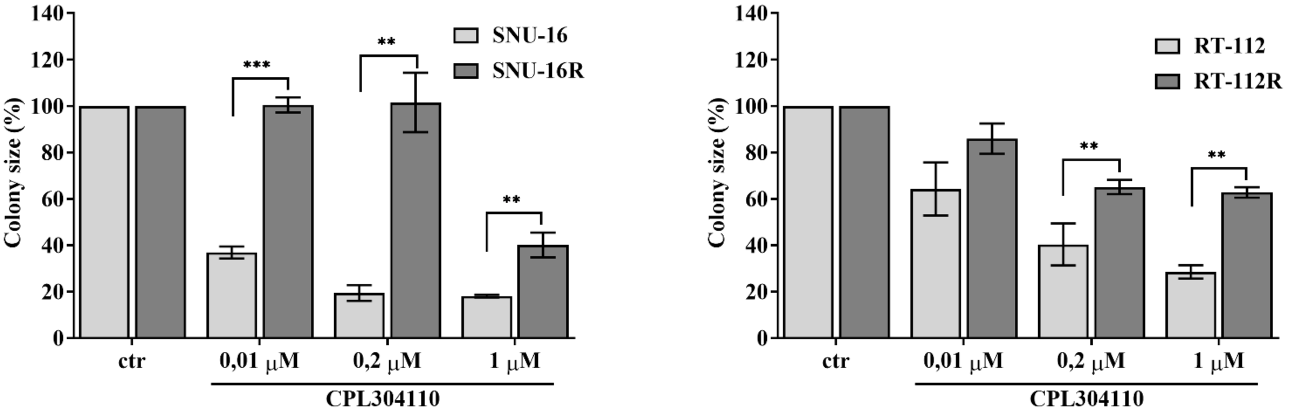

B

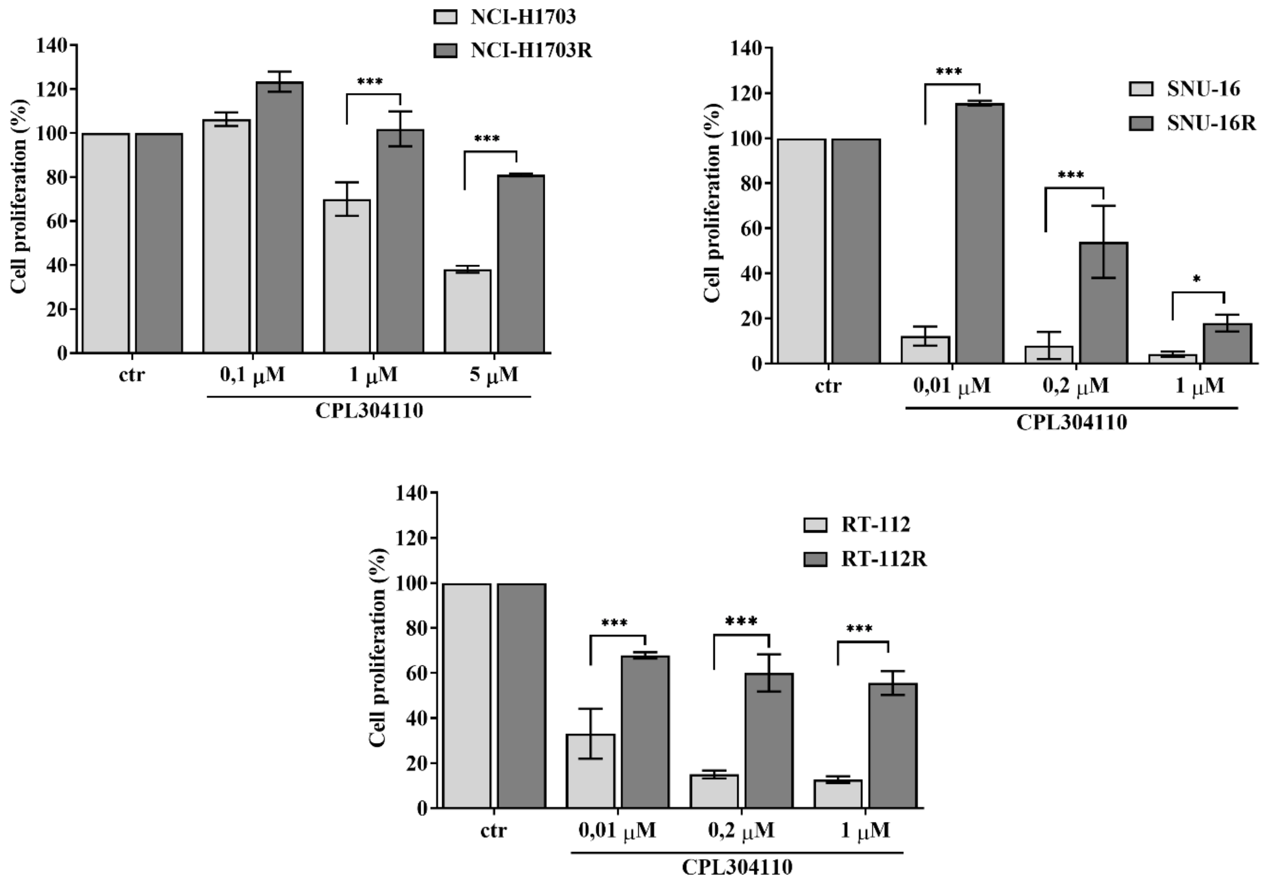

C

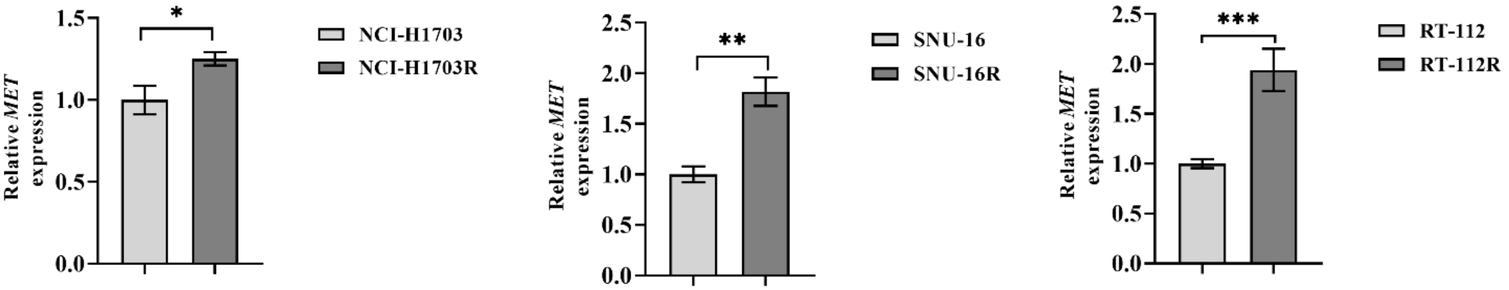

Figure S2. (A) Quantitative analysis of parental and CPL304110-resistant cells grown in 3D Matrigel® in the presence of CPL304110. Colonies were measured and statistically analysed with ImageJ. Data are expressed as mean ± SD, ** $p \leq 0.01$, *** $p \leq 0.001$, n = 3. **(B)** Proliferation analysis was evaluated by MTT in parental and resistant cells exposed to CPL304110 at the indicated concentrations for 96 h. Data are expressed as mean ± SD, * $p \leq 0.05$, ** $p \leq 0.01$, *** $p \leq 0.001$, n=3. **(C)** qPCR analysis of *MET* expression in parental and resistant cells. Data are expressed as mean ± SD, * $p \leq 0.05$, ** $p \leq 0.01$, *** $p \leq 0.001$, n=3.

Supplementary Figure 3.

A

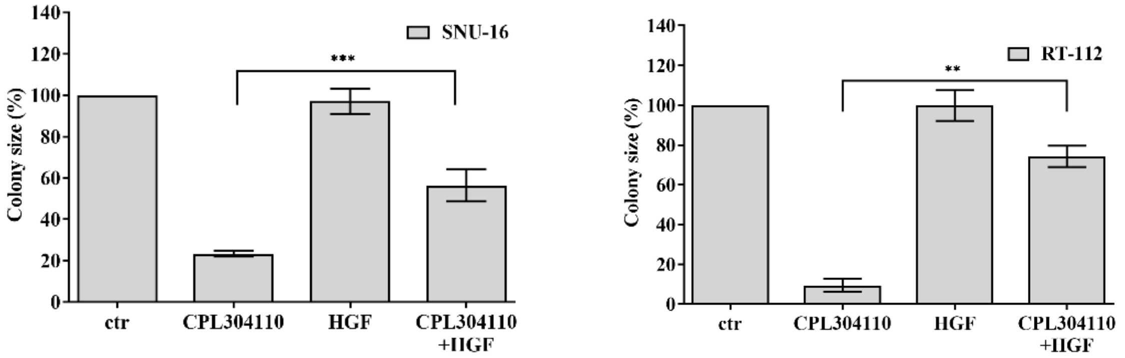

B

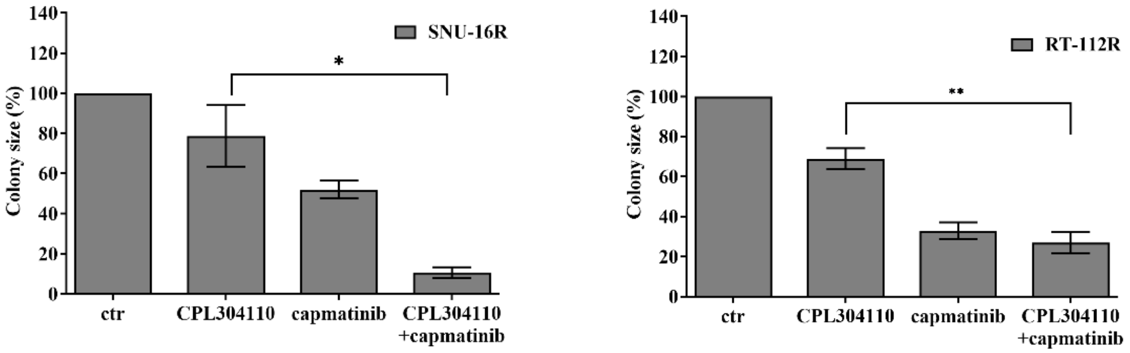

Figure S3. (A) Quantitative analysis of control cells grown in 3D Matrigel® in the presence of CPL304110 (1 µM) and/or HGF (50 ng/ml). **(B)** Quantitative analysis of CPL304110-resistant cells grown in 3D Matrigel® in the presence of CPL304110 (1 µM) and/or MET inhibitor - capmatinib (5 µM). Colonies were measured and statistically analysed with ImageJ. Data are expressed as mean ± SD, * $p \leq 0.05$, ** $p \leq 0.01$, *** $p \leq 0.001$, n=3.

Supplementary Figure 4.

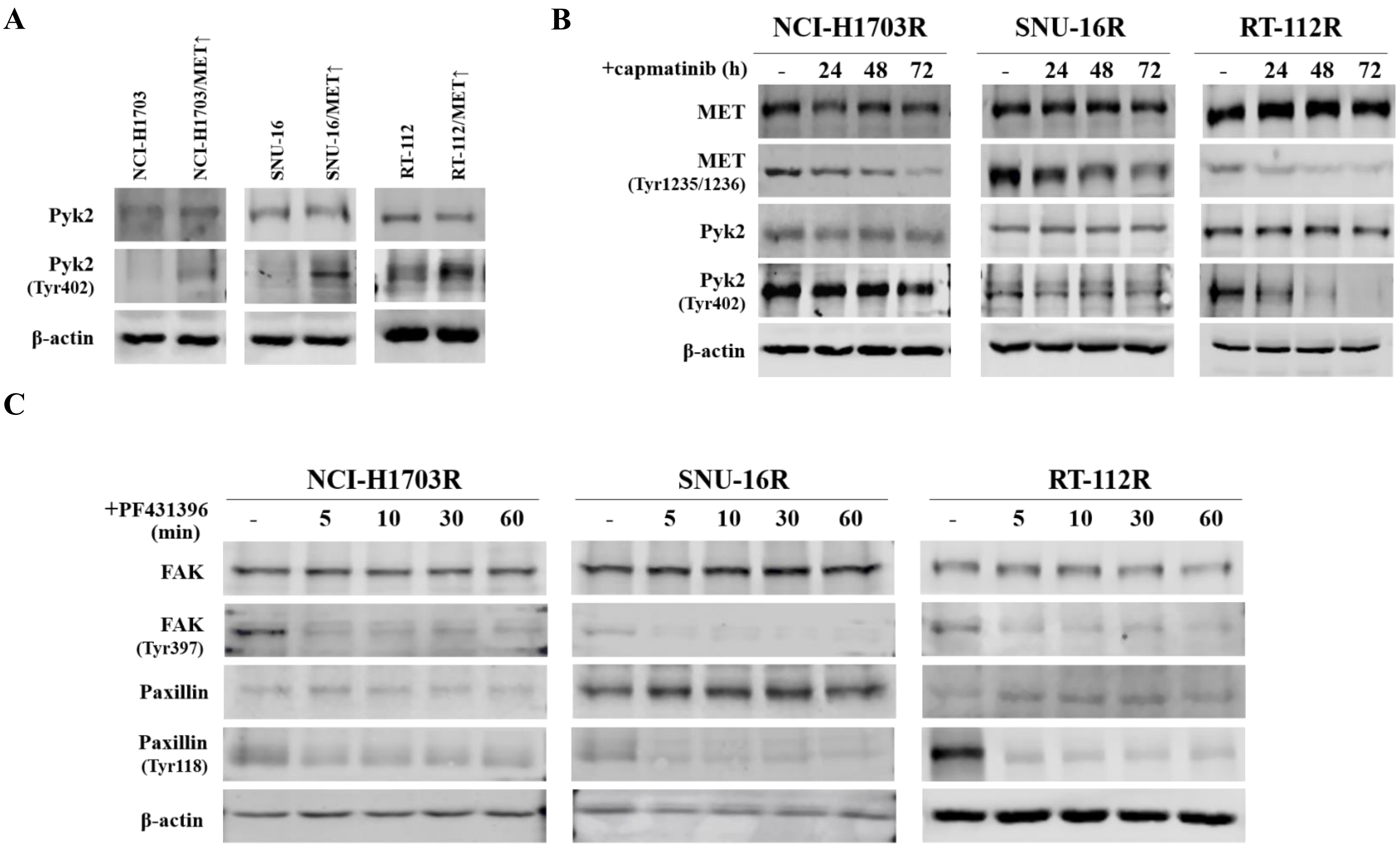

Figure S4. Western blot analysis was performed to **(A)** evaluated Pyk2 phosphorylation level in NCI-H1703, SNU-16 and RT-112 with ectopic expression of MET, **(B)** assess Pyk2 phosphorylation in resistant cells after capmatinib (5 μM) treatment in indicated time, **(C)** evaluate phosphorylation level of focal adhesion components in resistant cells in presence of PF431396 (100 nM). Experiments were conducted in triplicates.

Supplementary Figure 5.

A

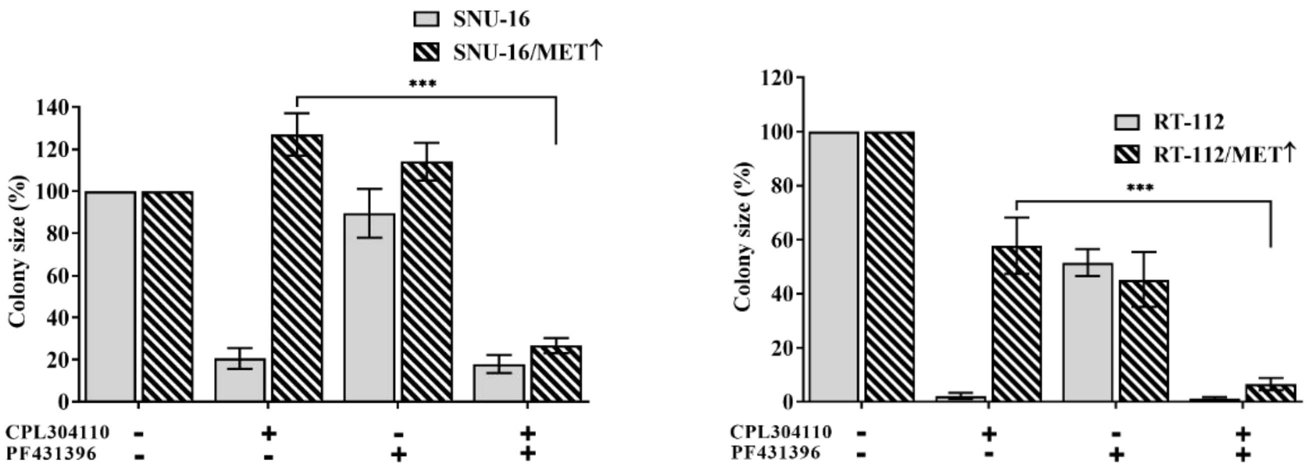

B

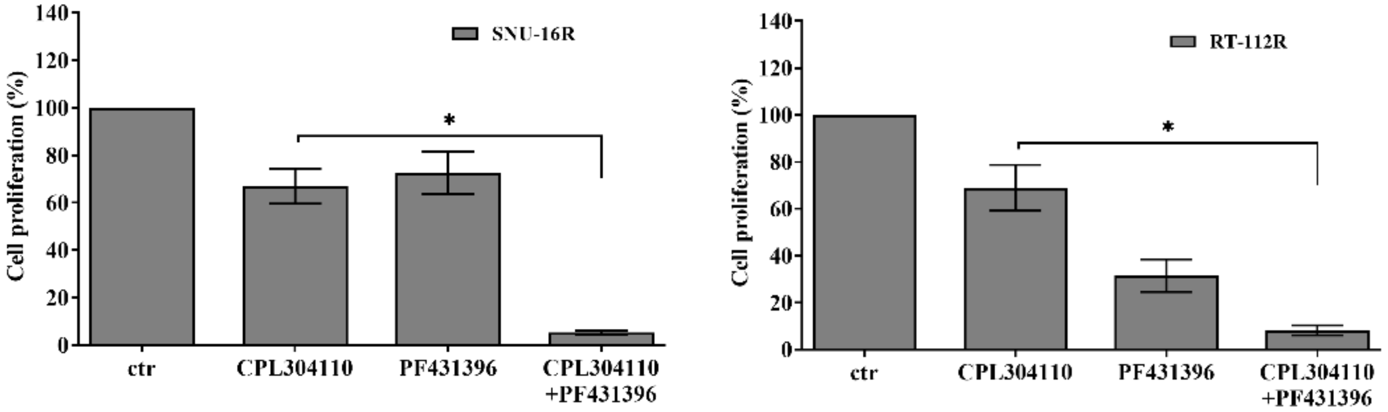

Figure S5. (A) Quantitative analysis of control and MET-overexpressing cells grown in 3D Matrigel® in the presence of CPL304110 (1 μM) and/or Pyk2 inhibitor - PF431396 (100 nM). Colonies were measured and statistically analysed with ImageJ. Data are expressed as mean ± SD, *** $p \leq 0.001$, n=3. **(B)** Proliferation analysis was evaluated by MTT in resistant cells exposed to (1 μM) and/or Pyk2 inhibitor - PF431396 (100 nM). Data are expressed as mean ± SD, * $p \leq 0.05$, *** $p \leq 0.001$, n=3.

Supplementary Figure 6.

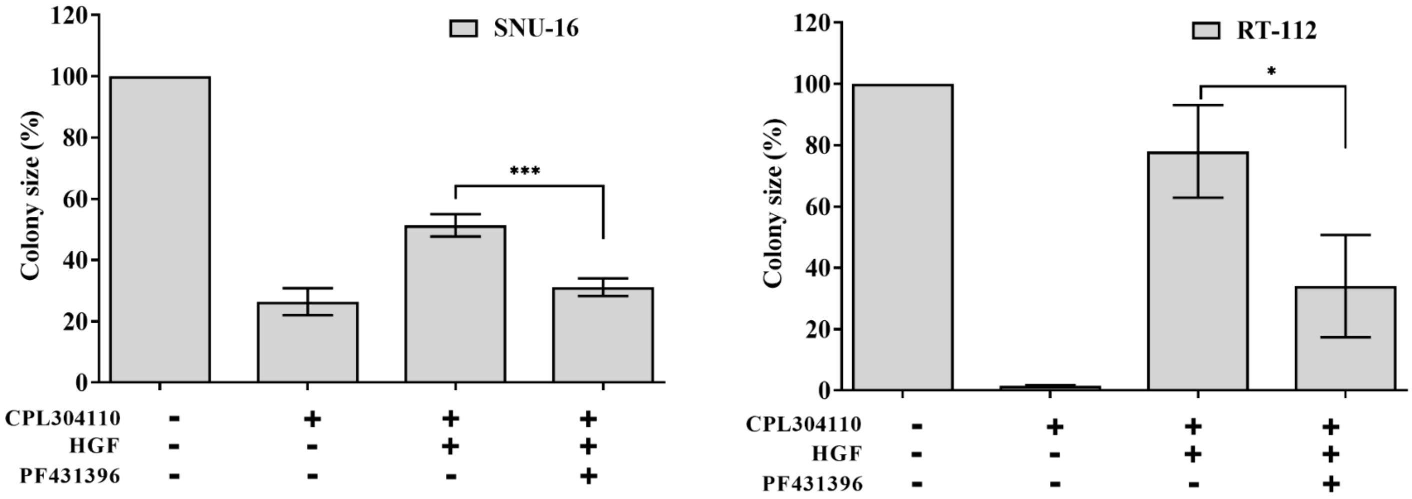

Figure S6. Quantitative analysis of NCI-H1703, SNU-16, RT-112 cells grown in 3D Matrigel® in the presence of CPL304110 (1 µM), HGF (50 ng/ml) and/or Pyk2 inhibitor - PF431396 (100 nM). Colonies were measured and statistically analysed with ImageJ. Data are expressed as mean ± SD, * $p \leq 0.05$, *** $p \leq 0.001$, n=3.

Supplementary Tables

Supplementary Table 1.

CELL LINE	Cancer	FGFR status	CPL304-110-01 IC50 [µM]
**DMS114**	small cell lung cancer	*FGFR1* amplification [4, 24]	1,5
**NCI-H1581**	non-small cell lung cancer	*FGFR1* amplification [4, 24]	0,025
**NCI-H1703**	squamous cell, non-small cell lung cancer	*FGFR1* amplification [4, 24]	1
**NCI-H2170**	squamous cell carcinoma, lung cancer	*no FGFR1* amplification [24]	15,4
**NCI-H520**	squamous cell carcinoma, lung cancer	*FGFR1* amplification [4, 24]	5,6
**SNU-16**	gastric carcinoma	*FGFR2* amplification [35]	0,04
**KATO III**	gastric carcinoma	*FGFR2* amplification [35]	0,04
**SNU-1**	gastric carcinoma	*no FGFR2* amplification [35]	1,6
**SNU-5**	gastric carcinoma	*no FGFR2* amplification [35]	1,2
**AGS**	gastric carcinoma	*no FGFR2* amplification [35]	1,1
**UM-UC-3**	urinary bladder transitional cell carcinoma	*FGFR3* wild type [31-33]	4,5
**UM-UC-14**	urinary bladder transitional cell carcinoma	*FGFR3* wild type [32], *FGFR3* mutation S249C [31, 32]	0,06
**UM-UC-16**	urinary bladder transitional cell carcinoma	*FGFR3* wild type [32], *FGFR3* mutation S249C [32]	3,1
**RT-112**	urinary bladder transitional cell carcinoma	*FGFR3* wild type [31, 32], *FGFR3* amplification [31], *FGFR3-TACC3* fusion [31, 33, 34]	0,15
**SW780**	urinary bladder transitional cell carcinoma	*FGFR3* wild type [31], *FGFR3* mutation S773F [31], *FGFR3-BAIAP2L1* fusion [31, 33, 34]	0,9
**639V**	ureter transitional cell carcinoma	*FGFR3* wild type [31], *FGFR3* mutation R248C [31, 33]	1,8
**T24**	urinary bladder transitional cell carcinoma	*FGFR3* wild type [31-34]	1,5

Table S1. CPL304110 IC_{50} was evaluated using the MTT assay in a panel of 17 cell lines derived from lung, gastric and bladder cancers.

Supplementary Table 2.

CELL LINE	Cancer	CPL304110 IC50 [µM]	AZD4547 IC50 [µM]
**NCI-H1703**	squamous cell, non-small cell lung cancer	1	3,4
**SNU-16**	gastric carcinoma	0,04	0,06
**RT-112**	urinary bladder transitional cell carcinoma	0,15	0,8

Table S2. IC_{50} comparison of FGFR inhibitors: CPL304110 vs. AZD4547 in NCI-H1703, SNU-16, RT-112 cells.
